# Health-related physical fitness, physical activity and its correlates among school going adolescents in hilly state in north India: a cross sectional survey

**DOI:** 10.1186/s12889-024-17808-3

**Published:** 2024-02-07

**Authors:** Ranjeeta Kumari, Bhola Nath, Yogesh Singh, Rupsha Mallick

**Affiliations:** 1 Present Address: All India Institute of Medical Sciences Rishikesh, Rishikesh, Uttarakhand 249203 India; 2 Present Address: All India Institute of Medical Sciences Raebareli, Raebareli, Uttar Pradesh 229405, India

**Keywords:** Physical fitness, Physical activity levels, Harvard step test, Health-related physical fitness, School going adolescents, Sit and reach test, Shoulder stretch test

## Abstract

**Introduction:**

Health-related physical fitness, which includes body composition, cardiorespiratory fitness, muscular endurance, flexibility, power, and strength are associated with risks of chronic diseases and promote good health and wellness. There have been reports of increasing levels of physical inactivity among children and adolescents, leading to increasing rates of obesity and decreased physical fitness. The present study was conducted among school going adolescents to estimate the levels and correlates of PF for timely intervention.

**Methodology:**

School based cross-sectional study was done among students of class 8-11th in Government schools of Garhwal division of Uttarakhand. Multistage stratified random sampling was applied for recruitment of study participants. We recruited a final sample size of 634 students. Validated questionnaires and standard methods for assessment of physical fitness, physical activity levels and other variables such as waist circumference, hip circumference, BMI and hemoglobin estimation were done.

**Results:**

Average and above average cardiorespiratory fitness score as per Harvard step test among boys (54.3%) was significantly higher as compared to girls (21.3%) (χ2 = 88.93, *p* < 0.001). There was a significant association between gender and dominant handgrip strength (χ2 = 8.02, *p* = 0.01) as well as between gender and Shoulder stretch test (SST) of dominant (χ2 = 17.5, *p* < 0.05) as well as nondominant arm (χ2 = 13.5, *p* < 0.05). Sit and reach test results also showed a significant association with gender (χ2 = 27.17, *p* < 0.001). Gender, hemoglobin level, BMI and PAL scores significantly predicted cardiorespiratory fitness scores (*R*2 = 0.188, *F* value of the model = 37.69, *p* =< 0.001)).

**Conclusion:**

Physical fitness of school going adolescents in Garhwal division of Uttarakhand was better than other parts of India, with significant gender differences. Physical activity levels (PAL) were poor and are also a significant predictor of physical fitness. More emphasis needs to be paid on the health and fitness of girl students. School based policies to increase PAL among students through innovation and rewards may go a long way in improving the long-term health of the students.

## Introduction

Physical fitness (PF) may be defined as the ability of the body systems to work together efficiently to allow a person to be healthy and perform activities of daily living with the least effort possible [1]. Physical fitness has two components: health-related and skill-related [1]. Health-related PF, which includes body composition, cardiorespiratory fitness, muscular endurance, flexibility, power, and strength are associated with risks of chronic diseases and promote good health and wellness [1].

PF has been reported to be an important issue from a public health perspective and has been found to be associated with various health related outcomes, including cardiovascular diseases, mental health, and skeletal health [2]. A decline in the levels of PF among both adults and adolescents has been observed over recent years [3–5]. It is also hypothesized that childhood PF is a predictor of PF in adulthood, which is a risk factor of cardiovascular diseases [6, 7]. With the increasing prevalence of non-communicable diseases in younger age groups, levels of PF are expected to have a stronger impact on the health outcomes in this age group as well [8, 9].

Various correlates of PF have been reported in different studies. Most commonly studied correlates of PF include overweight and obesity [10–13]. Physical activity (PA) has been reported as another important correlate of PF [14]. There have been reports of increasing levels of physical inactivity among children and adolescents, leading to increasing rates of obesity and decreased PF [15]. Both obesity and physical inactivity are affected by, as well as affect PF levels. Studies have reported significant rise in the level of physical inactivity and obesity among adolescents and other age groups in different parts of the world [14, 16–19].

The present study was conducted among school going adolescents to estimate the levels and correlates of PF. Adolescence is a phase of transitioning to university life, where the levels of both PF and PA are expected to decrease further, thereby increasing the risk for non-communicable diseases in coming years [16, 18, 20–22].

This assessment would provide us an opportunity to institute timely corrective measures for changing the health behaviour of these adolescents, who are in their habit-forming years.

We expected a higher level of PF and activity as compared to other states and regions in India, as Uttarakhand is a geographically hilly state and is a major contributor to Indian army, which is expected to motivate students to improve their PA and fitness [23].

The objectives of the study were to assess specific components of health-related PF viz. Cardiorespiratory fitness, Strength of muscles of upper part of the body and Flexibility among school going adolescents and to estimate the Physical activity levels (PAL) and assess the perceptions of students regarding advantages and barriers to Physical activity. We also intended to determine the association of PF and Physical activity levels (PALs) among study participants along with determination of significant predictors of PF using regression analysis.

## Material and methods

### Study design

The present study was a school-based cross-sectional study. The schools were selected using multistage stratified random sampling. Uttarakhand is a hilly state in north India and is composed of two divisions, namely Garhwal and Kumaon. The present study was conducted in Garhwal division of Uttarakhand. Each division is further classified into zones based on the altitude. One district was randomly chosen from each of the three zones, i.e., low, middle, and high altitude. From each of the districts one block was randomly chosen and one senior secondary school was chosen from each block. In each school, at least 50 students were selected randomly from class 8–11. The period of data collection was from August 2019 to September 2020. Ethics approval for conducting the study was obtained from the Institutional Ethics Committee of the institute (AIIMS/IEC/19/803). The study was in accordance with Indian Council of Medical Research guidelines in Human beings and adhered to principles of Good clinical practice and the Declaration of Helsinki.

### Participants

The present study was conducted among school-going adolescents in class 8-11^th^ in the Government schools of Garhwal division of Uttarakhand. **Inclusion criteria:** Students studying from 8 to 11^th^ class and those who received parental consent could participate in the study. **Exclusion criteria:** Students were screened by PA readiness questionnaire (PAR-Q) for their ability to undertake PA and were excluded if found ineligible. Physical activity readiness questionnaire (PAR-Q) contains seven questions, which screens for any factors which may prohibit PA such as presence of a heart condition or prescription from a doctor to refrain from physical activity; feeling of chest pain on doing physical activity; having a chest pain in past month when not physically active; loss of balance because of dizziness or loss of consciousness; presence of a bone or joint problem, which could worsen by PA; currently on medication for blood pressure or heart condition; any other reason which may requires refraining from physical activity. If the response to any of the seven questions was yes, the child was excluded [24]. Students were also screened for any bleeding disorder by asking a question “Have you ever continued bleeding for more than 1 min after you got a needle prick or very minor injury/ scratch?” A positive response or inability to answer the question also made the student ineligible to participate in the study. Inability to obtain consent from school/ parent/ children (anyone) was considered as an exclusion criteria. Also, children who were suffering from any disease in which PA was contraindicated or have been sick in the last week or have a bleeding disorder were excluded. **Sampling:** Multistage stratified random sampling was applied for recruitment of study participants as detailed under the heading of study design above. The number of students selected from each class and number of boys and girls was based on probability proportion to size. **Sample size:** Total number of students recruited in the study were:

$$\begin{array}{l}({\text{No}}.\mathrm{\, of\, students\, in \,each \,school})\mathrm{ \,X\, }(\mathrm{Number \,of \,schools \,in \,each \,district})\mathrm{ \,X \,}(\mathrm{number \,of \,districts})\\ = 50\mathrm{ \,X \,}4\mathrm{ \,X \,}3\\ = 600\mathrm{\, students}\end{array}$$

Taking a 5% non-response, we recruited a final sample size of 634 students.

### Data sources/ measurement

Various study tools used to achieve the objectives of current study included the use of questionnaires, anthropometry, PF tests and biochemical and physiological tests. A field investigator was recruited for the purpose of data collection and trained for all the aspects of data collection.

### Physical activity

For assessment of PA of the students, we used Physical Activity Questionnaire for Adolescents (PAQ-A) developed by Kowalski KC et al., which has a consistently high validity and moderate reliability [25, 26]. It is a self-reported questionnaire using a total of ten items. The questionnaire developed by Kowalski KC et al. had originally twenty-three different physical activities in first question. We used a modified version with only thirteen relevant activities as has been done by other researchers in India [27]. Composite score of first nine items was taken and mean scores were obtained for each student, which ranged from one to five. A score of one indicated low PA, and a score of five indicated high PA. Based on these scores, participants were classified as active or sedentary. Those who had a score of ≥ 3 were categorized as active while those with the scores < 3 were categorized as sedentary [28]. The last question enquired whether students participated in any PA during the previous week or not and it further explored the reasons for not participating in PA. This question was not used to score the PA level but used to present reasons for not participating in PA [15]. Anthropometric and physiological measurements were done using standard procedures as prescribed by WHO in the STEPS manual [29].

Height was measured in cms using a stadiometer by SECA. Standard procedure for height measurement was done. Weight was measured in Kg and gm using a Digital weighing scale. Hip and waist Circumference was measured in cms using Ergonomic circumference measuring tape Mechanical Make SECA. They were measured in a standing position. It was not feasible to undress the students for hip and waist circumference measurement. However, the students were asked to remove any sweaters during these measurements. Resting Blood pressure was measured using a standard electronic sphygmomanometer after the student was sitting comfortably on a chair for at least 5 min.

### Assessment of PF

PF was assessed using following methods:

1. Cardiorespiratory fitness was assessed using Harvard step test and time taken for heart rate recovery [30, 31].

The score was obtained from the formula.

$$\mathrm{Index \,of \,fitness }= (\mathrm{time \,of \,stepping \,in \,seconds\, x\, }100)/5.5\mathrm{ \,pulse \,count}.$$

The interpretation of score was as follows [32]:

- < 54 = Poor; 54–67 = Low Average; 68–82 = Average; 83–96 = Good; > 96 = Excellent

Classification of study participants according to VO2 max based on gender criteria was also done based on the VO2 max values of the Western population. Calculation of VO2 Max was done using following formulas [33]:

$$\begin{array}{l}{\text{Males}}:\mathrm{ \,VO}2\mathrm{ \,Max \,in \,ml}/{\text{kg}}/{\text{min}}= 111.33- \,(0.42\mathrm{ \,X \,Step\, test \,pulse \,rate},\mathrm{ \,beats}/\mathrm{ \,min})\\ {\text{Females}}:\mathrm{\, VO}2\mathrm{ \,Max \,in \,ml}/{\text{kg}}/\mathrm{min }= 65.81- \,(0.184\mathrm{ \,X \,Step \,test \,pulse \,rate},\mathrm{ \,beats}/\mathrm{ min})\end{array}$$

Strength of muscles of upper part of the body was assessed using Camry Electronic Hand grip dynamometer and recorded for dominant and non-dominant arm. Grip Strength Ratings for Males and females were classified according to Camry Electronic Hand Dynamometer Instruction manual available at Handgrip Strength Norms [34].

2. Flexibility was assessed using Shoulder stretch test and Sit and reach test [35, 36].

**Procedure for Shoulder stretch test:** To assess the dominant shoulder, the participant was asked to take his/her dominant hand over the dominant shoulder and down the back as if to pull up a zipper or scratch between the shoulder blades trying to touch the fingers of the nondominant-hand. The field investigator observed whether the fingers touch or not. Similar procedure was done for the non-dominant shoulder. If the student is able to touch the fingers with the dominant hand over the shoulder, a “Y” is recorded for the dominant side; otherwise, an “N” was recorded.

3. Procedure for conducting Sit and Reach Test [36]:

**Equipment:** Wooden scale, box, tape

The student performed Harvard step test first and then did the Sit and reach test. They were asked to remove their shoes. The investigator secured the ruler to the box top with the tape so that the front edge of the box lined up with the 15 cm (6 inches) mark on the ruler and the zero end of the ruler points towards the athlete. The student sat on the floor with their legs fully extended with the bottom of their bare feet against the box. The student was asked to place one hand on top of the other, slowly bend forward and reach along the top of the ruler as far as possible holding the stretch for two seconds. The investigator recorded the distance reached by the student’s fingertips in cm. The student performed the test three times. Best of all the values was recorded as final reading. Table 1 shows the normative data for 16 to 19-year-olds which was used for this test for the purpose of assessment [36, 37]:

**Table 1:** Gender wise normative data for assessment of sit and reach test results

Gender	Excellent	Above average	Average	Below average	Poor
Male	> 14	14.0 – 11.0	10.9 – 7.0	6.9 – 4.0	< 4
Female	> 15	15.0 – 12.0	11.9 – 7.0	6.9 – 4.0	< 4

Measurement of Hemoglobin was carried out by using a Hemoglobinometer (Hemocue 301) with a make of Boditech Med Inc. Standard procedures for sample collection, testing, interpretation and disposal as done in NFHS-4 were followed [38].

**Quality control during data collection:** A field investigator was recruited for the purpose of data collection and trained for all aspects of data collection. Supervisory visits were conducted by Principal investigator to monitor data collection operations to ensure that correct survey procedures were followed, and that data quality was maintained. Standardized and validated questionnaires, instruments and methods were used for data collection. The data was collected by the same investigator to overcome interviewer bias.

### Statistical analysis

Data was analyzed using IBM SPSS Statistics version 23, SPSS South Asia Pvt Limited, Bangalore, India. Quantitative variables were summarized as mean and SD or median where applicable. Normality analysis was carried out for quantitative variables using the Shapiro Wilk test. A *p* value of > 0.05 in Shapiro wilk test indicated normal distribution of sample data. However, comparison of means was done using parametric tests viz. independent t tests, as we assumed the distribution of data to be normal in study population from which the sample was obtained and the sample size was much greater than minimum sample size of 30 [39]. Independent variables included age and gender for the purpose of descriptive analysis. Dependent variables included PAL, PF results (VO2 Max, Handgrip strength, Sit and reach test results), perceived barriers and advantages of physical activity. Categorical variables were reported as proportions. For proportions, the Chi square test was applied to find out the association between independent and dependent variables. Multiple Linear regression analysis was carried out to find independent predictors of cardiorespiratory fitness and handgrip strength in dominant and non- dominant hand. A *p* value of less than 0.05 was considered significant. Wherever the expected cell frequency was less than five, Fischer exact test results were reported.

## Results

A total of 324 boys and 310 girls were recruited in the study. Seventy-one percent of the students belonged to nuclear family while rest of the students (29%) were from joint family.

Fathers of 525 (82.8%) students completed their education from primary up to the level of college/ university, while fathers of 77 (12.1%) students were illiterate or received less than primary school education. Rest of them either did not know/ refused (1.4%) or had lost their father (1.3%). Only 2.2% fathers were postgraduate. About 19% of the mothers had no formal schooling, while 33.4% had completed primary school education; only 5% had an undergraduate or postgraduate degree.

59.8% of students’ fathers were self-employed, while 28.9% and 8.5% who were non-government and government employee, respectively. Similarly, 84.4% of students’ mother was homemaker while 15.6% of mothers were engaged in different types of employment.

One hundred twenty-six students (19.9%) were from class 8 while around 27.9% of students were from class 9 and 10. Students from class 11 constituted 24.4% of all students. The mean age of students was 14.4 years, with a standard deviation of 1.4 years. The mean age of boys was 14.6 ± 1.4 years, while that of girls was 14.3 ± 1.3 years. The age of study participants ranged from 10 to 19 years.

It was observed that all boys had a recovery heart rate greater than 65 beats/ min with a predicted Vo2 max of 84.03 ml/kg/min or lower. The corresponding figures for girls was 60 and 54.77, respectively. Tests of normality for predicted VO_2_ Max. revealed that it was normally distributed among both boys and girls. Shapiro wilk test statistic for males was 0.995 and 0.996 and *p* value was 0.334 and 0.709 respectively, indicating normal distribution (Table 2).

**Table 2:** Gender wise comparison of recovery heart rate and VO_2_ max among study participants based on Harvard step test

**Percentile Ranking**	**Boys (*n* = 324)**		**Girls (*n* = 310)**	
	**Recovery HR (beats/ min.)**	**Predicted VO**_**2**_** Max. (ml/kg/min)**	**Recovery HR (beats/ min.)**	**Predicted VO**_**2**_** Max. (ml/kg/min)**
**100**	65	84.03	60	54.77
**95**	82	76.78	79	51.27
**90**	88	74.16	88	49.62
**85**	92	72.69	92	48.88
**80**	98	70.17	97	47.92
**75**	101	68.91	101	47.23
**70**	105	67.23	103	46.86
**65**	107	66.39	106	46.31
**60**	109	65.55	109	45.67
**55**	112	64.29	112	45.20
**50**	116	62.61	115	44.65
**45**	119	61.35	118	44.10
**40**	121	60.51	120	43.73
**35**	124	59.25	123	43.15
**30**	128	57.57	126	42.63
**25**	130	56.73	129	42.07
**20**	133	55.47	132	41.55
**15**	137	53.68	136	40.79
**10**	143	51.27	139	40.23
**5**	153	47.07	147	38.67

98% of male students as per VO2 Max norms were classified as having good and above category while all female students fell under good and above category [33, 40, 41]. The differences between boys and girls was observed to be statistically significant.

Proportion of boys (54.3%) having average and above average cardiorespiratory fitness score as per Harvard step test was significantly higher as compared to girls (21.3%) (χ2 = 88.93, *p* < 0.001). The proportion of boys (33.6%) with poor cardiorespiratory fitness as per Harvard test was almost half that of girls (69.7%) (Table 3).

**Table 3:** Comparison of Step test performance for cardiorespiratory fitness between boys and girls

	**Criteria for Boys**	**Criteria for Girls**	**Boys (*n* = 324)**		**Girls (*n* = 310)**		**Total (*n* = 634)**		**x**^**2**^** value, *p* value**
			No.	%	No.	%	No.	%	
**VO2 Max (Mean, SD)**			62.63, 8.91		44.71, 3.77				
**Classification of students as per VO**_**2**_** Max norms**									
Poor	33.0–36.4	23.60–28.9	2	0.6	-	-	2	0.3	6.85, 0.03
Fair	36.5–42.4	29.0–32.9	3	0.9	0		3	0.5	
Good	42.5–46.4	33.0–36.9	7	2.2	7	2.3	14	2.2	
Excellent	46.5–52.4	37.0–41.0	27	8.3	45	14.5	72	11.4	
Superior	> 52.4	> 41.0	285	88.0	258	83.2	543	85.6	
**Classification of cardiorespiratory fitness as per Harvard step test**									
Poor (< 54)			109	33.6	216	69.7	325	51.3	88.93, < 0.001
Low average & above (Score ≥ 54)			39	12.0	28	9.0	67	10.6	
Average (68–82)			48	14.8	25	8.1	73	11.5	
Good (83–96			67	20.7	23	7.4	67	14.2	
Excellent (> 96)			61	18.8	18	5.8	325	12.5	

The results of handgrip strength showed that 30.9% of boys had weak handgrip strength in dominant hand and 35.9% had weak strength in non-dominant hand. The corresponding figures for girls was 24.5% and 35.2% respectively. On the other hand, 33.6% and 29.9% of boys had strong handgrip strength in dominant and non-dominant hand respectively. Correspondingly, strong handgrip strength in dominant and non-dominant hand was found among 29% and 27.1% girls. There was a significant association between gender and dominant handgrip strength, whereas no significant association was observed for non-dominant handgrip strength and gender (Table 4).

**Table 4:** Hand grip strength ratings among boys and girls (in kg) in dominant hand and non-dominant hand

**AGE category in years**	**Weak**		**Normal**		**Strong**	
	**Dominant**	**Non-Dominant**	**Dominant**	**Non-Dominant**	**Dominant**	**Non-Dominant**
**Boys**						
12–13 yrs criteria	< 19.4		19.4–31.2		> 31.2	
**N (%)** (*n* = 74)	**17 (23)**	21 **(28.4)**	**33 (44.6)**	36 **(48.6)**	**24 (32.4)**	17 **(23.0)**
14–15 yrs criteria	< 28.5		28.5–44.3		> 44.3	
**N (%)** (*n* = 170)	**56 (32.9)**	66 **(38.8)**	**51 (30)**	47 **(27.6)**	**63(37.1)**	57 **(33.5)**
16–17 yrs criteria	< 32.6		32.6–52.4		> 52.4	
**N (%)** (*n* = 74)	**24 (32.4)**	26 **(35.1)**	**30(40.5)**	27 **(36.5)**	**20(27.0)**	21 **(28.4)**
18–19 yrs criteria	< 35.7		35.7–55.5		> 55.5	
**N (%)** (*n* = 6)	**3 (50)**	**3 (50)**	**1 (16.7)**	**1 (16.7)**	**2 (33.3)**	**2 (33.3)**
**Total boys (*n* = 324)** No. (%)	**100 (30.9)**	**116 (35.9)**	**115 (35.5)**	**111 (34.2)**	**109 (33.6)**	**97 (29.9)**
**Girls**						
**10–11** yrs** criteria**	**< 11.8**		**11.8–21.6**		**> 21.6**	
No. (%) **(*n* = 3)**	0 (0)	0	2 (66.7)	2 (66.7)	1(33.3)	1 (33.3)
**12–13** yrs **criteria**	**< 14.6**		**14.6–24.4**		**> 24.4**	
No. (%) **(*n* = 74)**	18 (24.3)	31 (41.9)	43 (58.1)	31(41.9)	13 (17.6)	12 (16.2)
**14–15 yrs criteria**	**< 15.5**		**15.5–27.3**		**> 27.3**	
No. (%) **(*n* = 176)**	44 (25)	61 (34.6)	83 (47.2)	72 (40.9)	49 (27.8)	43 (24.4)
**16–17 yrs criteria**	**< 17.2**		**17.2–29.0**		**> 29.0**	
No. (%) **(*n* = 54)**	12 (22.2)	15 (27.8)	16 (29.6)	12 (22.2)	26 (48.1)	27 (50)
**18–19 yrs criteria**	**< 19.2**		**19.2–31.0**		**> 31.0**	
No. (%) **(*n* = 3)**	2 (66.7)	2 (66.7)	0	0	1 (33.3)	1 (33.3)
**Total girls (*n* = 310)** No. (%)	**76 (24.5)**	**109 (35.2)**	**144 (46.5)**	**117 (37.7)**	**90 (29.0)**	**84 (27.1)**
**Total participants (*n* = 634)** No. (%)	**176 (27.8)**	**225 (35.5)**	**259 (40.8)**	**228 (35.9)**	**199 (31.3)**	**181 (28.5)**

Χ^2^ value, *p* value for comparison of handgrip strength in dominant hand between boys and girls: 8.02, 0.01; For non-dominant hand: 1.00, 0.606

The mean hand grip strength in dominant hand in boys and girls was 39.86 ± 22.25 kg and 23.93 ± 12.52 kg, respectively. The corresponding values for non- dominant hand were 38.55 ± 21.90 and 21.79 ± 11.67 kg respectively for boys and girls. The normality analysis of mean hand grip strength in dominant and non-dominant hand showed that it was not distributed normally with a *p* value of Shapiro wilk test being < 0.05 in both boys and girls.

**Fig. 1:**
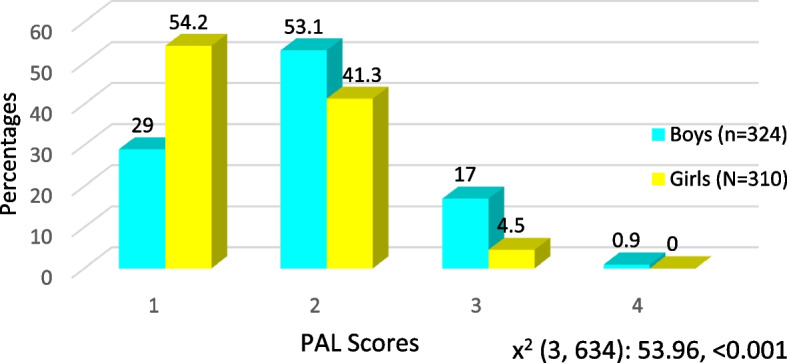
Gender wise comparison of physical activity level scores

A significant association was observed between gender and Shoulder stretch test (SST) of dominant (χ2 = 17.5, *p* < 0.05) as well as nondominant arm (χ2 = 13.5, *p* < 0.05). Sit and reach test results also showed a significant association with gender (χ2 = 27.17, *p* < 0.001), with a greater number of boys falling in excellent classification as compared to girls and higher proportion of girls with average results as compared to boys. It was also observed that proportion of boys (9.1%) falling in active category as per PAL scores was higher than that of girls (2.2%). The mean PAL scores in boys was also higher than that of girls (χ2 = 7.80, *p* < 0.001) (Table 5, Fig. 1). The normality analysis of sit and reach test results and mean PAL showed that it was not distributed normally with a *p* value of Shapiro wilk test being < 0.05 in both boys and girls.

**Table 5:** Comparison of Shoulder stretch test results, sit and reach test results and physical activity levels between boys and girls

**Test results**	**Boys (*N* = 324)**		**Girls (*N* = 310)**		**Test statistic**, *p* value
	No.	%	No.	%	
**Shoulder stretch test results**					
**Dominant hand**					
**Yes**	316	97.5	277	89.4	17.51, < 0.001
**No**	8	2.5	33	10.6	
**Non-dominant hand**					
**Yes**	260	80.2	209	67.4	13.5, < 0.001
**No**	64	19.7	101	32.6	
**Sit and reach test results**					
**Mean ± SD**	11.70 ± 3.08		11.58 ± 2.63		0.53, 0.59
**Classification (Criteria in cm)**					
**Excellent (> 14 Boys, > 15 girls)**^**a**^	70	21.6	26	8.4	27.17#, < 0.001
**Above average (14.0–11.0 boys, 15–12 girls)**	120	37.0	115	37.1	
**Average (10.9–7.0 boys, 11.9–7 girls)**^**a**^	120	37.0	157	50.6	
**Below average (6.9–4.00**	10	3.2	11	3.5	
**Poor (< 4)**	4	1.2	1	0.3	
**Classification of students based on PAL scores**					
**Sedentary PA (< 3)**	266	41.9	296	46.7	28.2, < 0.001
**Active (≥ 3)**	58	9.1	14	2.2	
**Physical activity levels (Mean, SD)**^**b**^	2.37, 0.65		2.00, 0.52		7.80, < 0.001

^#^Fisher exact test results

^a^Significant pairwise comparisons after applying Bonferroni correction

^b^Independent t test results

**Fig. 2:**
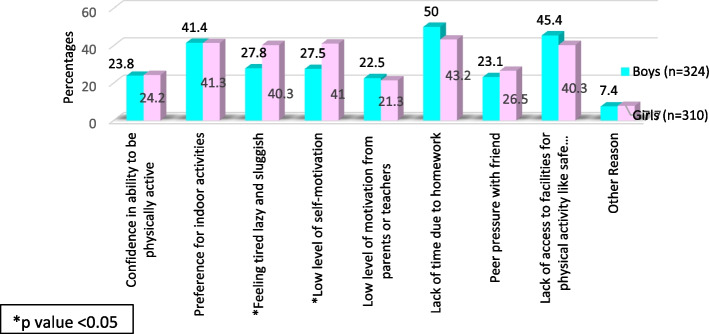
Gender wise comparison of perceived barriers of physical activity

The common perceived barriers of PA included lack of time due to homework (46.7%), lack of access to facilities for PA (42.9%) and preference for indoor activities (41.3%). The significantly larger proportion of girls reported specific barriers such as feeling tired, lazy, and sluggish (40.3%) and low level of self-motivation (41%) (Fig. 2).

**Fig. 3:**
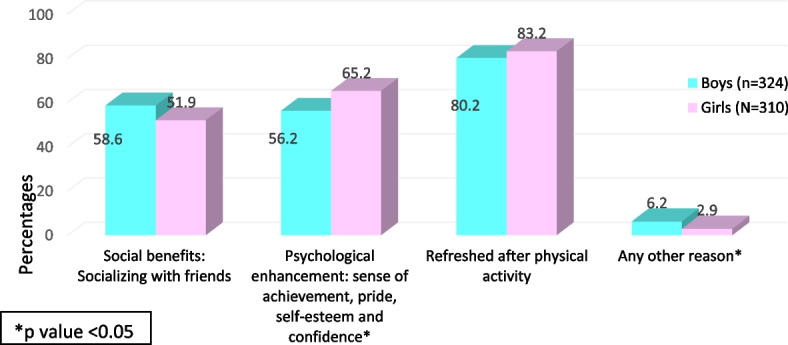
Advantages of physical activity as reported by students

Most of the students reported that the advantage of PA was that it refreshes them (81.7%). Also, they reported that it has social benefits (55.4%). Significantly larger number of girls reported that it provides psychological enhancement and provides a sense of achievement, pride, self-esteem, and confidence (χ2 = 5.36, *p* < 0.021) (Fig. 3).

Multiple linear regression was used to evaluate if gender, hemoglobin levels, BMI and PAL scores significantly predicted cardiorespiratory fitness scores. The fitted regression model was: Cardiorespiratory fitness score = 45.03- 0.22 (gender) + 0.21 (Hemoglobin level in mg/dl) -0.12(BMI in kg/m^2^) + 0.11 (PAL scores).

The overall regression was statistically significant (*R*^2^ = 0.188, *F* value of the model = 37.69, *p* =< 0.001). It was found that all the four predictor variables, i.e. gender, hemoglobin level, BMI and PAL scores significantly predicted cardiorespiratory fitness scores.

Similarly, it was observed that hemoglobin levels, PAL scores, age, gender, and cardiorespiratory fitness significantly predicted the handgrip strength in both dominant and non-dominant hand and the model was found to be statistically significant for both the outcomes. The model for determination of predictors of sit and reach test scores showed that BMI, hip circumference, Step test performance and Shoulder stretch test performance were significant predictors; however, age, gender and other variables were not found to be significant. The model was significant (*p* value =< 0.001), but the predictors explained only 7.6% variation (*R*^2^ = 0.076) (Table 6).

**Table 6:** Linear regression analysis to determine predictors of cardiorespiratory fitness scores, hand grip strength and sit and reach test scores

**Predictors**	**Unstandardized coefficients**	**Standardized coefficients**	**Sig**	**95% CI for B**	
	**B**	**Beta**		**Lower**	**Upper**
**For Cardiorespiratory fitness scores**					
Constant	45.03		< 0.001	19.80	70.27
Gender	-13.99	-0.22	< 0.001	-19.10	-8.87
Hemoglobin	3.26	.21	< 0.001	2.05	4.47
BMI	-1.32	-.12	0.001	-2.12	-.52
PAL score	5.47	.11	0.004	1.75	9.19
**Model Summary: *R*: 0.440, *R* Square: 0.188, *F* value of the model: 37.69, *p* value: < 0.001**					
**For Handgrip strength dominant hand**					
(Constant)	-30.94		.004	-51.75	-10.14
BMI	0.48	0.06	.096	-0.08	0.93
Haemoglobin	0.90	0.09	.019	0.15	1.66
Mean PA level	3.69	0.11	.002	1.38	6.01
Age	3.49	0.24	.000	2.48	4.50
Gender	-13.59	-0.34	.000	-16.82	-10.36
Step Test Category	1.14	0.09	.025	0.15	2.13
**Model Summary: *R*: 0.504, *R* Square: 0.254, *F* value of the model: 35.59, *p* value: < 0.001**					
**For Handgrip strength non-dominant hand**					
(Constant)	-34.28		.001	-54.37	-14.19
BMI	0.45	0.06	.068	-0.03	0.94
Haemoglobin	0.97	0.10	.009	0.24	1.71
Mean PA level	3.59	0.11	.002	1.36	5.83
Age	3.62	0.26	.000	2.64	4.59
Gender	-14.14	-0.36	.000	-17.26	-11.02
Step Test category	0.97	0.07	.047	0.01	1.93
**Model Summary: *R*: 0.533, *R* Square: 0.277, *F* value of the model: 41.52, *p* value: < 0.001**					
**For Sit and Reach test**					
(Constant)	6.20		0.000	3.07	9.33
BMI	0.16	0.16	0.017	0.03	0.30
Waist circumference	-0.04	-0.10	0.164	-0.09	0.02
Hip circumference	0.07	0.17	0.008	0.02	0.12
Step Test category	-0.28	-0.15	0.000	-0.44	-0.13
Shoulder stretch test dominant	-1.37	-0.12	0.003	-2.26	-0.47
Age	0.13	0.07	0.112	-0.03	0.30
Gender	0.05	0.01	0.864	-0.48	0.57
**Model Summary: *R*: 0.294, *R* Square: 0.076, *F* value of the model: 8.48, *p* value: < 0.001**					

## Discussion

The present study has attempted to comprehensively assess the school going adolescents with respect to their Physical fitness and Physical activity levels, both of which are correlated to each other and are considered to be important modifiable predictors of current and future health of an individual [42].

The Health-related PF assessment of study participants in the present study showed that Cardiorespiratory fitness of most of the students was excellent to superior as per the VO2 max norms based on recovery heart rate [33]. However, as per the Harvard step test results, most of the students scored poorly with a score of < 54, with significant differences between boys and girls. These findings are consistent with our hypothesis of better physical fitness as compared to other states in India such as Rajasthan, which is a non-mountainous state in western India, where a study showed that the mean VO2 max was 45.30 ± 7.35 mL/kg/min for males and 35.71 ± 5.29 mL/kg/min for females, whereas in the present study it was observed to be 62.63 ± 8.91 and 44.71 ± 3.77, respectively, which was quite higher [40]. This difference may be attributed to the hilly terrain, which is expected to improve the cardiorespiratory fitness of the residents and the younger age group in present study. Also, the assessment in the present study was done using Harvard step test, whereas the study in Rajasthan used direct method with the help of a gas analyser. Another study conducted in Imphal, a mountainous region showed that the mean VO2 max was 41.3471 ± 6.80 ml/kg/min, which was also lower than that reported in the present study [43]. However, with respect to Harvard test results, another study done in Nagpur, reported that only 18.05% had ‘poor score’ and most of the students (66.37%) had average scores, while the present study reported that most of the students had poor score (51.3%) and only 11.5% had average score [44]. The better cardiorespiratory fitness observed among males is also consistent with the results in these studies [40, 43, 44]. Various physiological mechanisms have been attributed by various researchers attributing the gender wise difference of cardiorespiratory fitness to body composition especially fat percentage, blood hemoglobin concentration and heart size [45, 46].

It has been reported by several researchers that muscular strength is inversely associated with premature mortality from all causes, suicide and cardiovascular diseases in young adulthood and cancer [47–50].

The results obtained in the present study with respect to handgrip strength is mostly consistent with the findings in other studies conducted in different parts of the world [51–53]. As in previous studies, we observed that the handgrip strength in males was better than females in both dominant and non-dominant hand [51–53]. However, the mean hand grip strength of boys in the present study was higher than students of similar age in Spain, while that of girls was observed to be lower, which may be attributed to ethnic, geographical and anthropometric differences [53]. Another study conducted in Brazil, found that mean handgrip strength among boys and girls in pubescent age group was 413.9 ± 13.8 Newton (42.2 ± 1.4 kg) and 393.2 ± 13.8 Newton (40.09 ± 1.4 kg), which was higher than that reported in the present study [54]. A study conducted on students of similar age group at New York, showed that the mean handgrip strength in dominant hand in boys was lower (37.66 ± 7.50) to that reported in present study (39.86 ± 22.25). However mean handgrip strength in dominant hand among girls (28.71 ± 5.10 kg) was higher than that reported in the present study (23.93 ± 12.52 kg) [55]. Similar results were also shown by HELENA study conducted in 10 European countries, which reported a mean strength of 31.2 ± 6.4 kg in boys and 26.1 ± 5.1 kg in girls [56]. A study conducted in Chilean adolescents showed that the mean handgrip strength was lower among boys (31.5 kg), while it was similar to that of present study among girls (24.0 kg) [57].

As in previous studies, we also observed that grip strength was higher in dominant hand as compared to non-dominant hand [51, 53, 58, 59]. In contrast to previous studies, we observed that BMI was not a significant correlate of handgrip strength as seen in the regression analysis [53, 60].

We also observed that hemoglobin level was a significant modifiable predictor of handgrip strength. This is an important finding which could help us design interventions to improve the handgrip strength among students. Also, positive association with cardiorespiratory fitness suggests that improving one measure of physical fitness promotes the other aspects also [54].

With respect to sit and reach test, it was observed that the students in present study were less flexible than students in other countries as mean sit and reach results were only 11.70 and 11.58 cm in boys and girls respectively as compared to 27.4 and 32.2 cm respectively in Chinese adolescents [61]. Results from HELENA study also reported similar results with the best mean sit and reach results on a back saver Sit and reach test as 19.1 cm and 26.2 cm in boys and girls respectively [56]. Similarly another study done among Portuguese adolescents also showed higher readings as compared to the present study [62]. All these studies also found a significant difference between boys and girls with respect to sit and reach test results [56, 61, 62]. However, we did not observe significant difference in the mean values; but on categorization of students based on the sit and reach test values, we found that boys had better scores than girls, which was in contrast to other studies done in China, Europe and Portugal. However, on regression analysis, gender was not found to be a significant predictor; only BMI, hip circumference, shoulder stretch test and step test performance were observed to be significant predictors of sit and reach scores.

The shoulder stretch test results were found to be satisfactory with most of the students (93.5%) being able to do it with dominant hand, with the corresponding proportion being lower with non-dominant hand (74%), which was as per the expectation based on physiological reasoning. Another study also showed that the participants performed better with right arm as compared to left arm (mean distance between two arms being 0.60 cm with right arm vs 3.60 cm with left arm) [63]. However, the lower proportion reported amongst girls was surprising as girls were expected to be more flexible as compared to the boys, as has been observed in studies reporting sit and reach test results also [56, 61, 62].

The status of physical activity among students in the present study was also found to be unsatisfactory with the mean levels in both boys and girls falling below the cut-off of 3, with girls having even lower levels than boys. This is consistent with the findings in other studies conducted in India and other countries which have reported declining trends in PAL [27, 64, 65]. It was strange to know that despite the advantages stated by the students, their involvement in PAL was low. The common barriers stated in the present study such as lack of time, motivation and academic stress are similar to that reported in another study and needs to be addressed through change in school policies as the schools serve as a good place for focussed group interventions for healthy lifestyle [27, 66, 67].

## Limitation

The present study has good external and internal validity as random sampling was done for selection of schools and students. Also, standard methods of measurement of various variables, which could be done in the field survey were used in the study. However, PAL was based on the responses of the students and would have been more accurate if objective methods of measurements such as accelerometer or pedometer were used. Nevertheless, the questionnaire used was validated for Indian settings and was administered by the same field investigator to avoid bias. Another limitation of the study was that it was conducted in one of the two regions of Uttarakhand. However, since the geographical characteristics of both the regions are similar, the findings of the study can be applied to another region of Uttarakhand also.

## Conclusion

The present study was a novel attempt to do a comprehensive assessment of health-related physical fitness among school going adolescents in this hilly region of India and has provided us with valuable insights into the distribution characteristics of each component as well as its modifiable predictors such as PAL, hemoglobin level, and BMI.

## Practical implications and future line of research

The study results show us that more emphasis needs to be paid on the health and fitness of girl students and focus on physical activity, weight management and anaemia correction for achieving better PF among students. School based policies to increase PAL among students through innovation and rewards may go a long way in improving the long-term health of the students [68, 69]. Further implementation research on interventions for improving health-related physical fitness through school-based interventions are planned to be undertaken. Also, qualitative research will be undertaken to understand the reasons for inadequate physical activity among students for designing appropriate interventions.

## Data Availability

The datasets generated and/or analysed during the current study are not publicly available currently as they have identifiers and also because the publication of papers from the research is under process. The data can be made available by the corresponding author on reasonable request.
